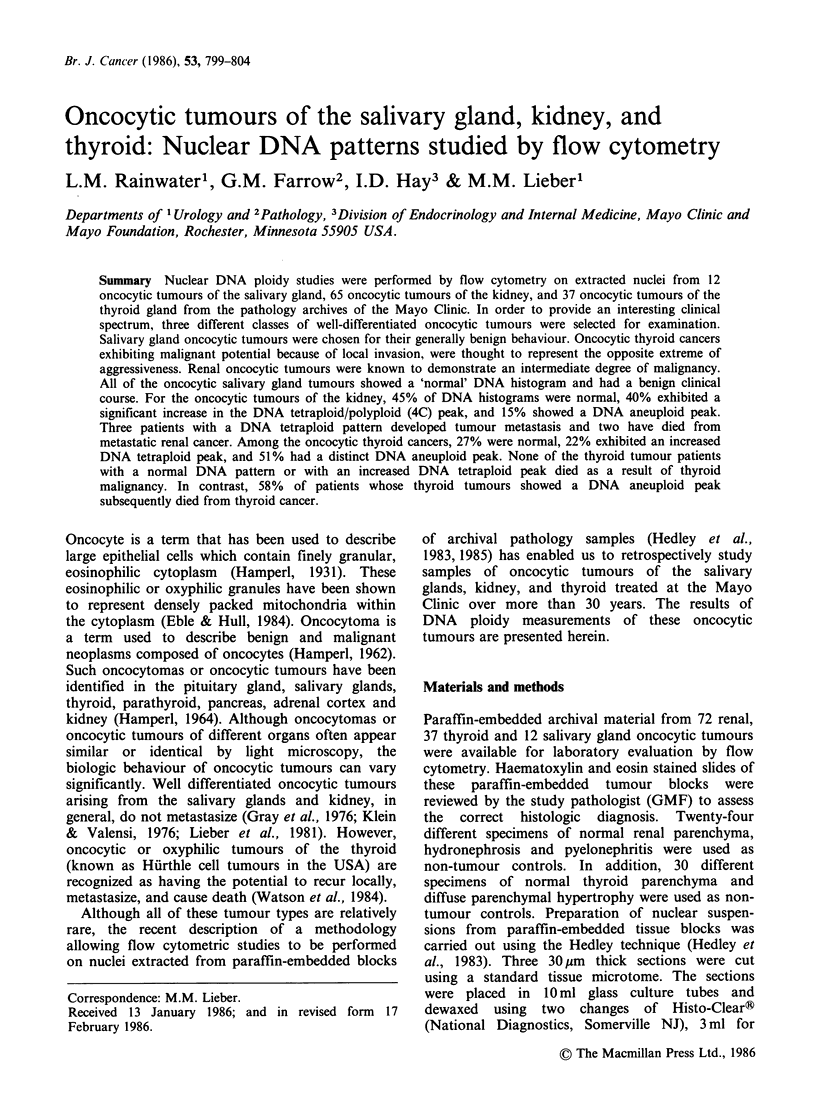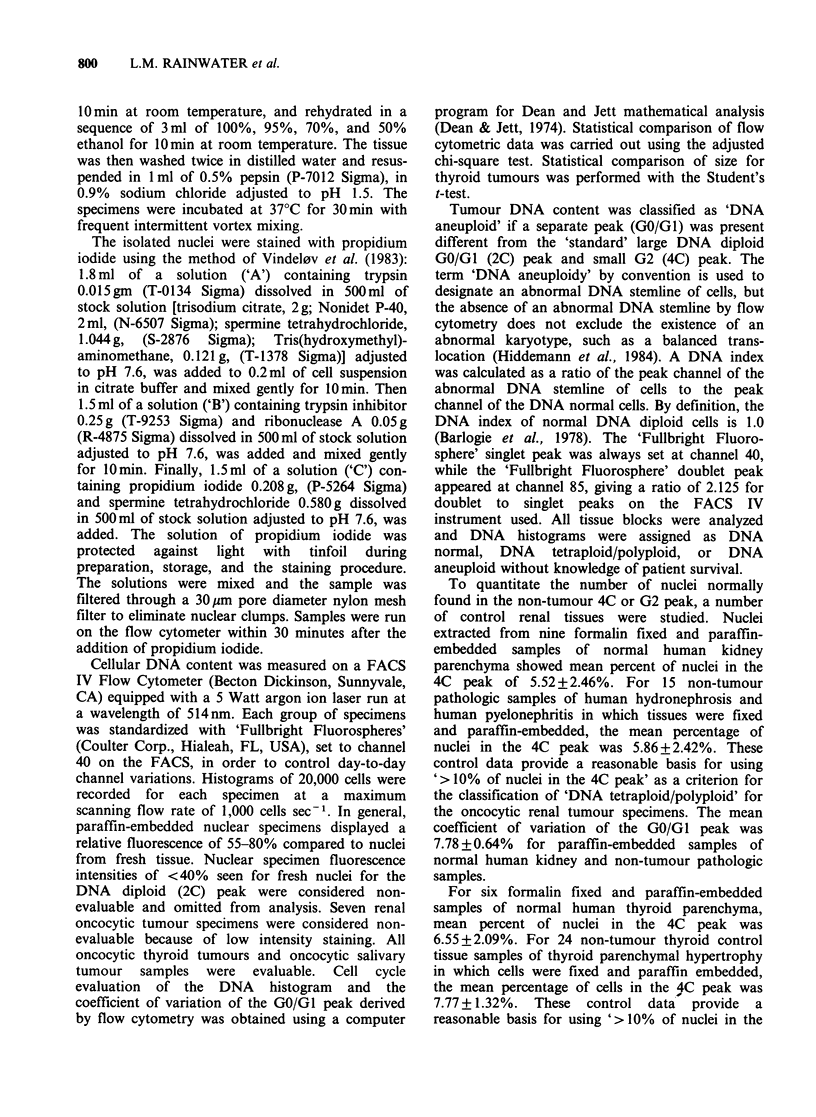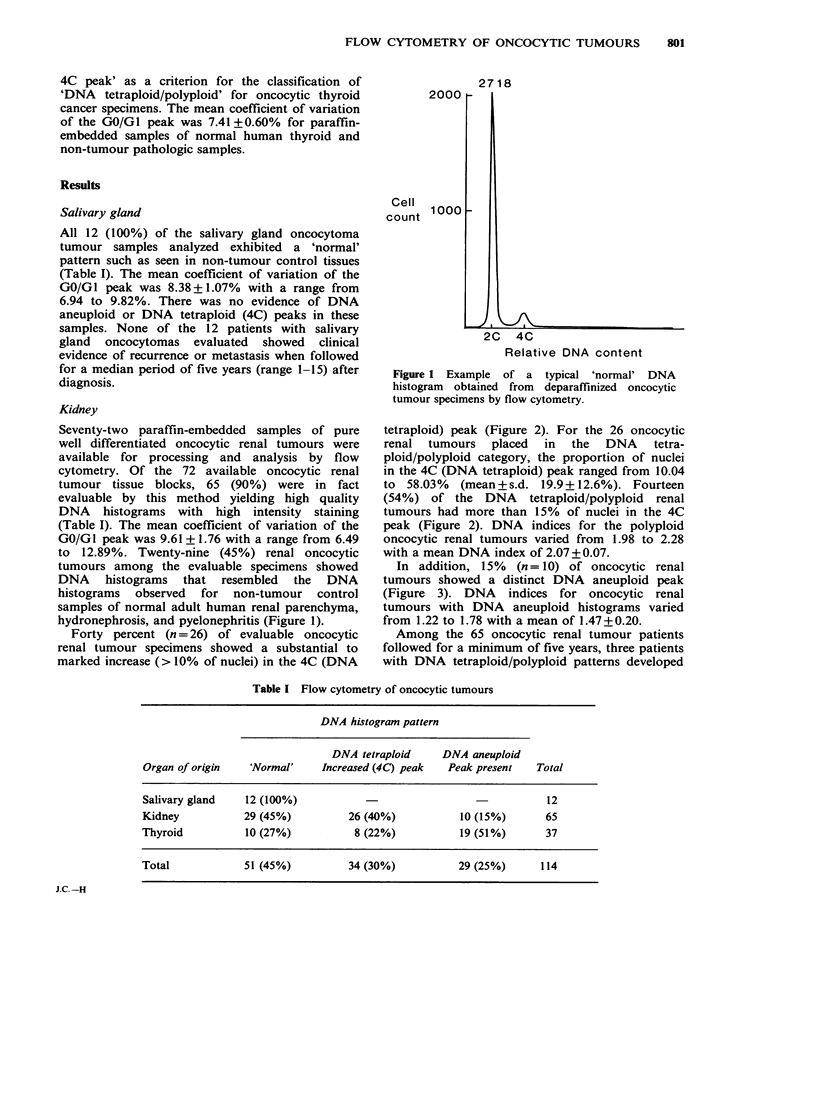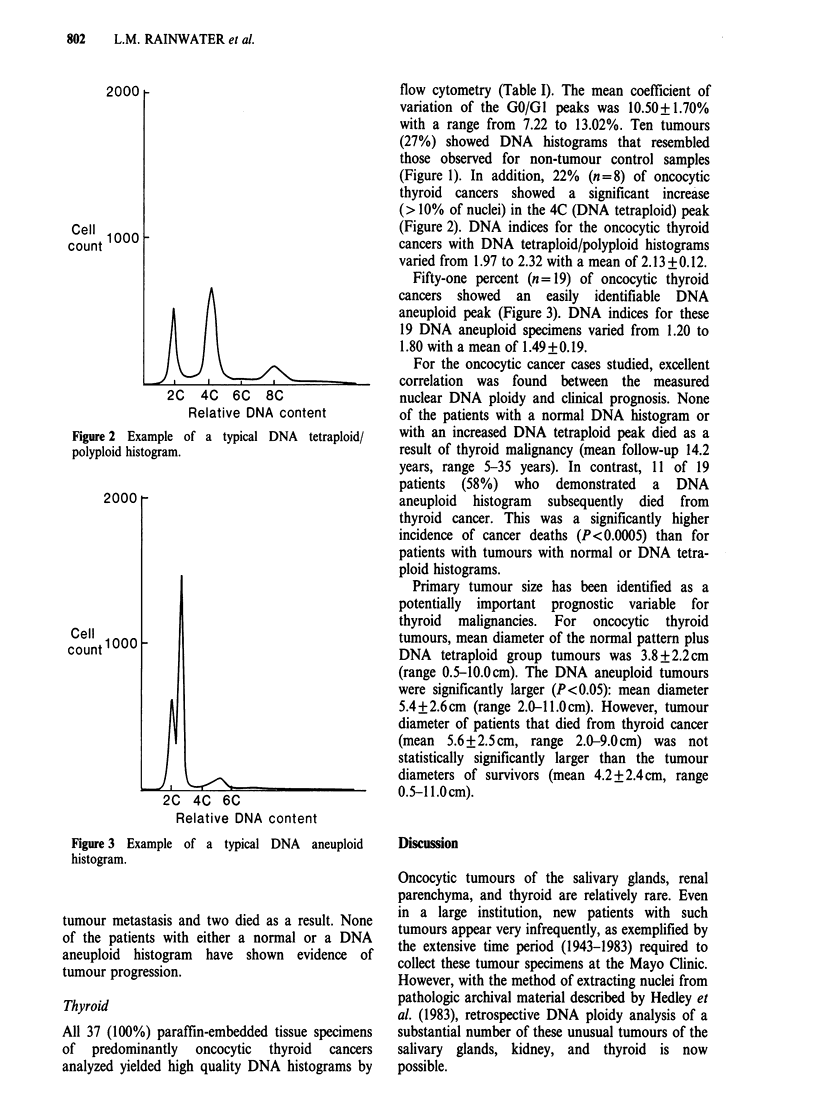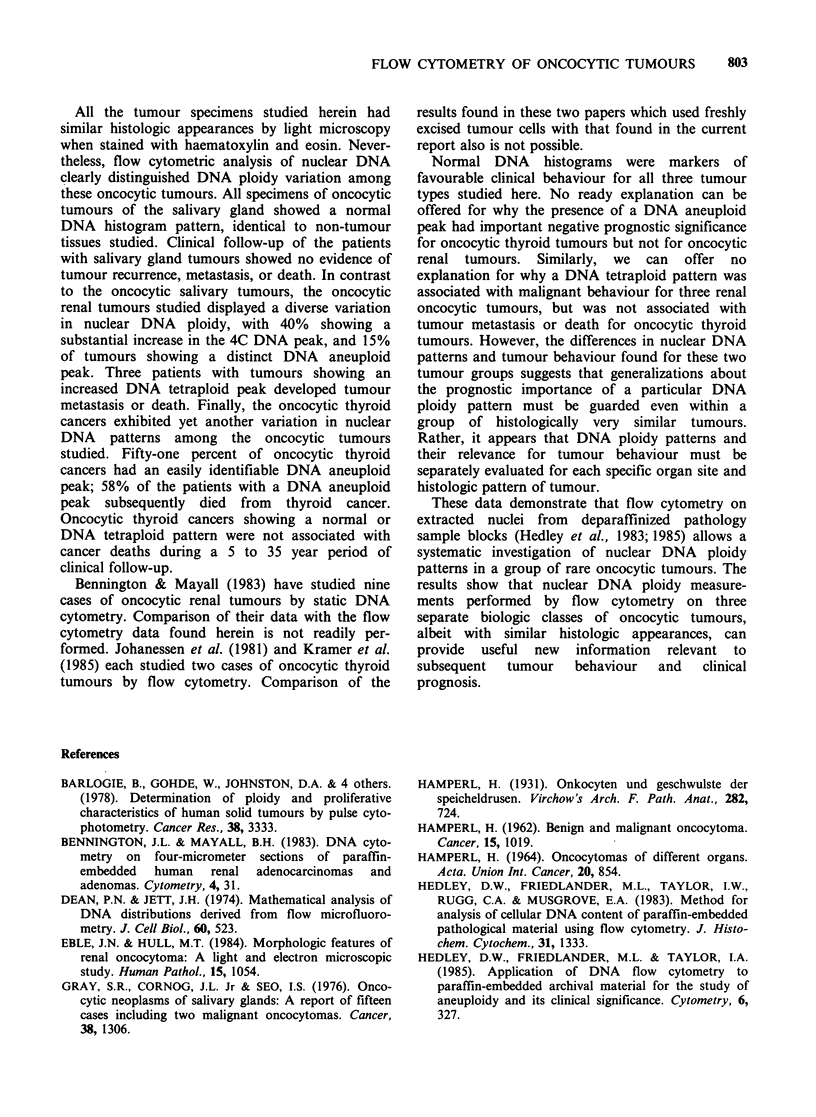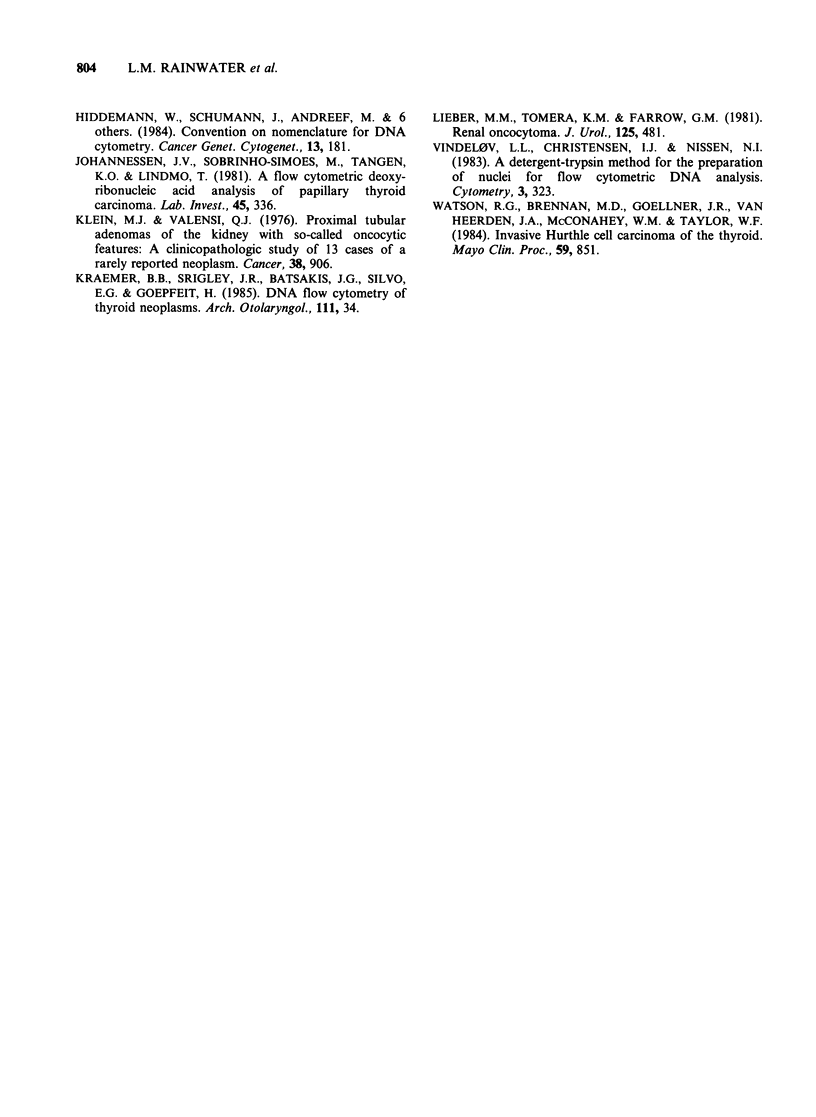# Oncocytic tumours of the salivary gland, kidney, and thyroid: nuclear DNA patterns studied by flow cytometry.

**DOI:** 10.1038/bjc.1986.135

**Published:** 1986-06

**Authors:** L. M. Rainwater, G. M. Farrow, I. D. Hay, M. M. Lieber

## Abstract

Nuclear DNA ploidy studies were performed by flow cytometry on extracted nuclei from 12 oncocytic tumours of the salivary gland, 65 oncocytic tumours of the kidney, and 37 oncocytic tumours of the thyroid gland from the pathology archives of the Mayo Clinic. In order to provide an interesting clinical spectrum, three different classes of well-differentiated oncocytic tumours were selected for examination. Salivary gland oncocytic tumours were chosen for their generally benign behaviour. Oncocytic thyroid cancers exhibiting malignant potential because of local invasion, were thought to represent the opposite extreme of aggressiveness. Renal oncocytic tumours were known to demonstrate an intermediate degree of malignancy. All of the oncocytic salivary gland tumours showed a 'normal' DNA histogram and had a benign clinical course. For the oncocytic tumours of the kidney, 45% of DNA histograms were normal, 40% exhibited a significant increase in the DNA tetraploid/polyploid (4C) peak, and 15% showed a DNA aneuploid peak. Three patients with a DNA tetraploid pattern developed tumour metastasis and two have died from metastatic renal cancer. Among the oncocytic thyroid cancers, 27% were normal, 22% exhibited an increased DNA tetraploid peak, and 51% had a distinct DNA aneuploid peak. None of the thyroid tumour patients with a normal DNA pattern or with an increased DNA tetraploid peak died as a result of thyroid malignancy. In contrast, 58% of patients whose thyroid tumours showed a DNA aneuploid peak subsequently died from thyroid cancer.


*Br. J. Cancer* (1986), **53**, 799–804

### Oncocytic tumours of the salivary gland, kidney, and thyroid: Nuclear DNA patterns studied by flow cytometry

L.M. Rainwater[1], G.M. Farrow[2], I.D. Hay[3] & M.M. Lieber[1]

*Departments of [1]Urology and [2]Pathology, [3]Division of Endocrinology and Internal Medicine, Mayo Clinic and Mayo Foundation, Rochester, Minnesota 55905 USA.*

**Summary** Nuclear DNA ploidy studies were performed by flow cytometry on extracted nuclei from 12 oncocytic tumours of the salivary gland, 65 oncocytic tumours of the kidney, and 37 oncocytic tumours of the thyroid gland from the pathology archives of the Mayo Clinic. In order to provide an interesting clinical spectrum, three different classes of well-differentiated oncocytic tumours were selected for examination. Salivary gland oncocytic tumours were chosen for their generally benign behaviour. Oncocytic thyroid cancers exhibiting malignant potential because of local invasion, were thought to represent the opposite extreme of aggressiveness. Renal oncocytic tumours were known to demonstrate an intermediate degree of malignancy. All of the oncocytic salivary gland tumours showed a 'normal' DNA histogram and had a benign clinical course. For the oncocytic tumours of the kidney, 45% of DNA histograms were normal, 40% exhibited a significant increase in the DNA tetraploid/polyploid (4C) peak, and 15% showed a DNA aneuploid peak. Three patients with a DNA tetraploid pattern developed tumour metastasis and two have died from metastatic renal cancer. Among the oncocytic thyroid cancers, 27% were normal, 22% exhibited an increased DNA tetraploid peak, and 51% had a distinct DNA aneuploid peak. None of the thyroid tumour patients with a normal DNA pattern or with an increased DNA tetraploid peak died as a result of thyroid malignancy. In contrast, 58% of patients whose thyroid tumours showed a DNA aneuploid peak subsequently died from thyroid cancer.

Oncocyte is a term that has been used to describe large epithelial cells which contain finely granular, eosinophilic cytoplasm (Hamperl, 1931). These eosinophilic or oxyphilic granules have been shown to represent densely packed mitochondria within the cytoplasm (Eble & Hull, 1984). Oncocytoma is a term used to describe benign and malignant neoplasms composed of oncocytes (Hamperl, 1962). Such oncocytomas or oncocytic tumours have been identified in the pituitary gland, salivary glands, thyroid, parathyroid, pancreas, adrenal cortex and kidney (Hamperl, 1964). Although oncocytomas or oncocytic tumours of different organs often appear similar or identical by light microscopy, the biologic behaviour of oncocytic tumours can vary significantly. Well differentiated oncocytic tumours arising from the salivary glands and kidney, in general, do not metastasize (Gray *et al.*, 1976; Klein & Valensi, 1976; Lieber *et al.*, 1981). However, oncocytic or oxyphilic tumours of the thyroid (known as Hürthle cell tumours in the USA) are recognized as having the potential to recur locally, metastasize, and cause death (Watson *et al.*, 1984).

Although all of these tumour types are relatively rare, the recent description of a methodology allowing flow cytometric studies to be performed on nuclei extracted from paraffin-embedded blocks of archival pathology samples (Hedley *et al.*, 1983, 1985) has enabled us to retrospectively study samples of oncocytic tumours of the salivary glands, kidney, and thyroid treated at the Mayo Clinic over more than 30 years. The results of DNA ploidy measurements of these oncocytic tumours are presented herein.

Correspondence: M.M. Lieber.
Received 13 January 1986; and in revised form 17 February 1986.

### Materials and methods

Paraffin-embedded archival material from 72 renal, 37 thyroid and 12 salivary gland oncocytic tumours were available for laboratory evaluation by flow cytometry. Haematoxylin and eosin stained slides of these paraffin-embedded tumour blocks were reviewed by the study pathologist (GMF) to assess the correct histologic diagnosis. Twenty-four different specimens of normal renal parenchyma, hydronephrosis and pyelonephritis were used as non-tumour controls. In addition, 30 different specimens of normal thyroid parenchyma and diffuse parenchymal hypertrophy were used as non-tumour controls. Preparation of nuclear suspensions from paraffin-embedded tissue blocks was carried out using the Hedley technique (Hedley *et al.*, 1983). Three 30 $\mu$m thick sections were cut using a standard tissue microtome. The sections were placed in 10 ml glass culture tubes and dewaxed using two changes of Histo-Clear® (National Diagnostics, Somerville NJ), 3 ml for

© The Macmillan Press Ltd., 1986

10 min at room temperature, and rehydrated in a sequence of 3 ml of 100%, 95%, 70%, and 50% ethanol for 10 min at room temperature. The tissue was then washed twice in distilled water and resuspended in 1 ml of 0.5% pepsin (P-7012 Sigma), in 0.9% sodium chloride adjusted to pH 1.5. The specimens were incubated at 37°C for 30 min with frequent intermittent vortex mixing.

The isolated nuclei were stained with propidium iodide using the method of Vindeløv *et al.* (1983): 1.8 ml of a solution ('A') containing trypsin 0.015 gm (T-0134 Sigma) dissolved in 500 ml of stock solution [trisodium citrate, 2 g; Nonidet P-40, 2 ml, (N-6507 Sigma); spermine tetrahydrochloride, 1.044 g, (S-2876 Sigma); Tris(hydroxymethyl)-aminomethane, 0.121 g, (T-1378 Sigma)] adjusted to pH 7.6, was added to 0.2 ml of cell suspension in citrate buffer and mixed gently for 10 min. Then 1.5 ml of a solution ('B') containing trypsin inhibitor 0.25 g (T-9253 Sigma) and ribonuclease A 0.05 g (R-4875 Sigma) dissolved in 500 ml of stock solution adjusted to pH 7.6, was added and mixed gently for 10 min. Finally, 1.5 ml of a solution ('C') containing propidium iodide 0.208 g, (P-5264 Sigma) and spermine tetrahydrochloride 0.580 g dissolved in 500 ml of stock solution adjusted to pH 7.6, was added. The solution of propidium iodide was protected against light with tinfoil during preparation, storage, and the staining procedure. The solutions were mixed and the sample was filtered through a 30 $\mu$m pore diameter nylon mesh filter to eliminate nuclear clumps. Samples were run on the flow cytometer within 30 minutes after the addition of propidium iodide.

Cellular DNA content was measured on a FACS IV Flow Cytometer (Becton Dickinson, Sunnyvale, CA) equipped with a 5 Watt argon ion laser run at a wavelength of 514 nm. Each group of specimens was standardized with 'Fullbright Fluorospheres' (Coulter Corp., Hialeah, FL, USA), set to channel 40 on the FACS, in order to control day-to-day channel variations. Histograms of 20,000 cells were recorded for each specimen at a maximum scanning flow rate of 1,000 cells $sec^{-1}$. In general, paraffin-embedded nuclear specimens displayed a relative fluorescence of 55–80% compared to nuclei from fresh tissue. Nuclear specimen fluorescence intensities of $<40\%$ seen for fresh nuclei for the DNA diploid (2C) peak were considered non-evaluable and omitted from analysis. Seven renal oncocytic tumour specimens were considered non-evaluable because of low intensity staining. All oncocytic thyroid tumours and oncocytic salivary tumour samples were evaluable. Cell cycle evaluation of the DNA histogram and the coefficient of variation of the G0/G1 peak derived by flow cytometry was obtained using a computer program for Dean and Jett mathematical analysis (Dean & Jett, 1974). Statistical comparison of flow cytometric data was carried out using the adjusted chi-square test. Statistical comparison of size for thyroid tumours was performed with the Student's *t*-test.

Tumour DNA content was classified as 'DNA aneuploid' if a separate peak (G0/G1) was present different from the 'standard' large DNA diploid G0/G1 (2C) peak and small G2 (4C) peak. The term 'DNA aneuploidy' by convention is used to designate an abnormal DNA stemline of cells, but the absence of an abnormal DNA stemline by flow cytometry does not exclude the existence of an abnormal karyotype, such as a balanced translocation (Hiddemann *et al.*, 1984). A DNA index was calculated as a ratio of the peak channel of the abnormal DNA stemline of cells to the peak channel of the DNA normal cells. By definition, the DNA index of normal DNA diploid cells is 1.0 (Barlogie *et al.*, 1978). The 'Fullbright Fluorosphere' singlet peak was always set at channel 40, while the 'Fullbright Fluorosphere' doublet peak appeared at channel 85, giving a ratio of 2.125 for doublet to singlet peaks on the FACS IV instrument used. All tissue blocks were analyzed and DNA histograms were assigned as DNA normal, DNA tetraploid/polyploid, or DNA aneuploid without knowledge of patient survival.

To quantitate the number of nuclei normally found in the non-tumour 4C or G2 peak, a number of control renal tissues were studied. Nuclei extracted from nine formalin fixed and paraffin-embedded samples of normal human kidney parenchyma showed mean percent of nuclei in the 4C peak of $5.52 \pm 2.46\%$. For 15 non-tumour pathologic samples of human hydronephrosis and human pyelonephritis in which tissues were fixed and paraffin-embedded, the mean percentage of nuclei in the 4C peak was $5.86 \pm 2.42\%$. These control data provide a reasonable basis for using '$>10\%$ of nuclei in the 4C peak' as a criterion for the classification of 'DNA tetraploid/polyploid' for the oncocytic renal tumour specimens. The mean coefficient of variation of the G0/G1 peak was $7.78 \pm 0.64\%$ for paraffin-embedded samples of normal human kidney and non-tumour pathologic samples.

For six formalin fixed and paraffin-embedded samples of normal human thyroid parenchyma, mean percent of nuclei in the 4C peak was $6.55 \pm 2.09\%$. For 24 non-tumour thyroid control tissue samples of thyroid parenchymal hypertrophy in which cells were fixed and paraffin embedded, the mean percentage of cells in the 4C peak was $7.77 \pm 1.32\%$. These control data provide a reasonable basis for using '$>10\%$ of nuclei in the

4C peak' as a criterion for the classification of 'DNA tetraploid/polyploid' for oncocytic thyroid cancer specimens. The mean coefficient of variation of the G0/G1 peak was 7.41±0.60% for paraffin-embedded samples of normal human thyroid and non-tumour pathologic samples.

### Results

### *Salivary gland*

All 12 (100%) of the salivary gland oncocytoma tumour samples analyzed exhibited a 'normal' pattern such as seen in non-tumour control tissues (Table I). The mean coefficient of variation of the G0/G1 peak was 8.38±1.07% with a range from 6.94 to 9.82%. There was no evidence of DNA aneuploid or DNA tetraploid (4C) peaks in these samples. None of the 12 patients with salivary gland oncocytomas evaluated showed clinical evidence of recurrence or metastasis when followed for a median period of five years (range 1–15) after diagnosis.

### *Kidney*

Seventy-two paraffin-embedded samples of pure well differentiated oncocytic renal tumours were available for processing and analysis by flow cytometry. Of the 72 available oncocytic renal tumour tissue blocks, 65 (90%) were in fact evaluable by this method yielding high quality DNA histograms with high intensity staining (Table I). The mean coefficient of variation of the G0/G1 peak was 9.61±1.76 with a range from 6.49 to 12.89%. Twenty-nine (45%) renal oncocytic tumours among the evaluable specimens showed DNA histograms that resembled the DNA histograms observed for non-tumour control samples of normal adult human renal parenchyma, hydronephrosis, and pyelonephritis (Figure 1).

Forty percent ($n=26$) of evaluable oncocytic renal tumour specimens showed a substantial to marked increase (>10% of nuclei) in the 4C (DNA tetraploid) peak (Figure 2). For the 26 oncocytic renal tumours placed in the DNA tetraploid/polyploid category, the proportion of nuclei in the 4C (DNA tetraploid) peak ranged from 10.04 to 58.03% (mean±s.d. 19.9±12.6%). Fourteen (54%) of the DNA tetraploid/polyploid renal tumours had more than 15% of nuclei in the 4C peak (Figure 2). DNA indices for the polyploid oncocytic renal tumours varied from 1.98 to 2.28 with a mean DNA index of 2.07±0.07.

In addition, 15% ($n=10$) of oncocytic renal tumours showed a distinct DNA aneuploid peak (Figure 3). DNA indices for oncocytic renal tumours with DNA aneuploid histograms varied from 1.22 to 1.78 with a mean of 1.47±0.20.

Among the 65 oncocytic renal tumour patients followed for a minimum of five years, three patients with DNA tetraploid/polyploid patterns developed

**Figure 1** Example of a typical 'normal' DNA histogram obtained from deparaffinized oncocytic tumour specimens by flow cytometry.

**Table I** Flow cytometry of oncocytic tumours

| *Organ of origin* | *DNA histogram pattern* 'Normal' | *DNA tetraploid Increased (4C) peak* | *DNA aneuploid Peak present* | *Total* |
|---|---|---|---|---|
| Salivary gland | 12 (100%) | — | — | 12 |
| Kidney | 29 (45%) | 26 (40%) | 10 (15%) | 65 |
| Thyroid | 10 (27%) | 8 (22%) | 19 (51%) | 37 |
| Total | 51 (45%) | 34 (30%) | 29 (25%) | 114 |

**Figure 2** Example of a typical DNA tetraploid/polyploid histogram.

**Figure 3** Example of a typical DNA aneuploid histogram.

tumour metastasis and two died as a result. None of the patients with either a normal or a DNA aneuploid histogram have shown evidence of tumour progression.

*Thyroid*

All 37 (100%) paraffin-embedded tissue specimens of predominantly oncocytic thyroid cancers analyzed yielded high quality DNA histograms by flow cytometry (Table I). The mean coefficient of variation of the G0/G1 peaks was $10.50 \pm 1.70\%$ with a range from 7.22 to 13.02%. Ten tumours (27%) showed DNA histograms that resembled those observed for non-tumour control samples (Figure 1). In addition, 22% ($n=8$) of oncocytic thyroid cancers showed a significant increase (>10% of nuclei) in the 4C (DNA tetraploid) peak (Figure 2). DNA indices for the oncocytic thyroid cancers with DNA tetraploid/polyploid histograms varied from 1.97 to 2.32 with a mean of $2.13 \pm 0.12$.

Fifty-one percent ($n=19$) of oncocytic thyroid cancers showed an easily identifiable DNA aneuploid peak (Figure 3). DNA indices for these 19 DNA aneuploid specimens varied from 1.20 to 1.80 with a mean of $1.49 \pm 0.19$.

For the oncocytic cancer cases studied, excellent correlation was found between the measured nuclear DNA ploidy and clinical prognosis. None of the patients with a normal DNA histogram or with an increased DNA tetraploid peak died as a result of thyroid malignancy (mean follow-up 14.2 years, range 5–35 years). In contrast, 11 of 19 patients (58%) who demonstrated a DNA aneuploid histogram subsequently died from thyroid cancer. This was a significantly higher incidence of cancer deaths ($P<0.0005$) than for patients with tumours with normal or DNA tetraploid histograms.

Primary tumour size has been identified as a potentially important prognostic variable for thyroid malignancies. For oncocytic thyroid tumours, mean diameter of the normal pattern plus DNA tetraploid group tumours was $3.8 \pm 2.2$ cm (range 0.5–10.0 cm). The DNA aneuploid tumours were significantly larger ($P<0.05$): mean diameter $5.4 \pm 2.6$ cm (range 2.0–11.0 cm). However, tumour diameter of patients that died from thyroid cancer (mean $5.6 \pm 2.5$ cm, range 2.0–9.0 cm) was not statistically significantly larger than the tumour diameters of survivors (mean $4.2 \pm 2.4$ cm, range 0.5–11.0 cm).

### Discussion

Oncocytic tumours of the salivary glands, renal parenchyma, and thyroid are relatively rare. Even in a large institution, new patients with such tumours appear very infrequently, as exemplified by the extensive time period (1943–1983) required to collect these tumour specimens at the Mayo Clinic. However, with the method of extracting nuclei from pathologic archival material described by Hedley et al. (1983), retrospective DNA ploidy analysis of a substantial number of these unusual tumours of the salivary glands, kidney, and thyroid is now possible.

All the tumour specimens studied herein had similar histologic appearances by light microscopy when stained with haematoxylin and eosin. Nevertheless, flow cytometric analysis of nuclear DNA clearly distinguished DNA ploidy variation among these oncocytic tumours. All specimens of oncocytic tumours of the salivary gland showed a normal DNA histogram pattern, identical to non-tumour tissues studied. Clinical follow-up of the patients with salivary gland tumours showed no evidence of tumour recurrence, metastasis, or death. In contrast to the oncocytic salivary tumours, the oncocytic renal tumours studied displayed a diverse variation in nuclear DNA ploidy, with 40% showing a substantial increase in the 4C DNA peak, and 15% of tumours showing a distinct DNA aneuploid peak. Three patients with tumours showing an increased DNA tetraploid peak developed tumour metastasis or death. Finally, the oncocytic thyroid cancers exhibited yet another variation in nuclear DNA patterns among the oncocytic tumours studied. Fifty-one percent of oncocytic thyroid cancers had an easily identifiable DNA aneuploid peak; 58% of the patients with a DNA aneuploid peak subsequently died from thyroid cancer. Oncocytic thyroid cancers showing a normal or DNA tetraploid pattern were not associated with cancer deaths during a 5 to 35 year period of clinical follow-up.

Bennington & Mayall (1983) have studied nine cases of oncocytic renal tumours by static DNA cytometry. Comparison of their data with the flow cytometry data found herein is not readily performed. Johanessen *et al.* (1981) and Kramer *et al.* (1985) each studied two cases of oncocytic thyroid tumours by flow cytometry. Comparison of the results found in these two papers which used freshly excised tumour cells with that found in the current report also is not possible.

Normal DNA histograms were markers of favourable clinical behaviour for all three tumour types studied here. No ready explanation can be offered for why the presence of a DNA aneuploid peak had important negative prognostic significance for oncocytic thyroid tumours but not for oncocytic renal tumours. Similarly, we can offer no explanation for why a DNA tetraploid pattern was associated with malignant behaviour for three renal oncocytic tumours, but was not associated with tumour metastasis or death for oncocytic thyroid tumours. However, the differences in nuclear DNA patterns and tumour behaviour found for these two tumour groups suggests that generalizations about the prognostic importance of a particular DNA ploidy pattern must be guarded even within a group of histologically very similar tumours. Rather, it appears that DNA ploidy patterns and their relevance for tumour behaviour must be separately evaluated for each specific organ site and histologic pattern of tumour.

These data demonstrate that flow cytometry on extracted nuclei from deparaffinized pathology sample blocks (Hedley *et al.*, 1983; 1985) allows a systematic investigation of nuclear DNA ploidy patterns in a group of rare oncocytic tumours. The results show that nuclear DNA ploidy measurements performed by flow cytometry on three separate biologic classes of oncocytic tumours, albeit with similar histologic appearances, can provide useful new information relevant to subsequent tumour behaviour and clinical prognosis.

### References

BARLOGIE, B., GOHDE, W., JOHNSTON, D.A. & 4 others. (1978). Determination of ploidy and proliferative characteristics of human solid tumours by pulse cytophotometry. *Cancer Res.*, **38**, 3333.

BENNINGTON, J.L. & MAYALL, B.H. (1983). DNA cytometry on four-micrometer sections of paraffin-embedded human renal adenocarcinomas and adenomas. *Cytometry*, **4**, 31.

DEAN, P.N. & JETT, J.H. (1974). Mathematical analysis of DNA distributions derived from flow microfluorometry. *J. Cell Biol.*, **60**, 523.

EBLE, J.N. & HULL, M.T. (1984). Morphologic features of renal oncocytoma: A light and electron microscopic study. *Human Pathol.*, **15**, 1054.

GRAY, S.R., CORNOG, J.L. Jr & SEO, I.S. (1976). Oncocytic neoplasms of salivary glands: A report of fifteen cases including two malignant oncocytomas. *Cancer*, **38**, 1306.

HAMPERL, H. (1931). Onkocyten und geschwulste der speicheldrusen. *Virchow's Arch. F. Path. Anat.*, **282**, 724.

HAMPERL, H. (1962). Benign and malignant oncocytoma. *Cancer*, **15**, 1019.

HAMPERL, H. (1964). Oncocytomas of different organs. *Acta. Union Int. Cancer*, **20**, 854.

HEDLEY, D.W., FRIEDLANDER, M.L., TAYLOR, I.W., RUGG, C.A. & MUSGROVE, E.A. (1983). Method for analysis of cellular DNA content of paraffin-embedded pathological material using flow cytometry. *J. Histochem. Cytochem.*, **31**, 1333.

HEDLEY, D.W., FRIEDLANDER, M.L. & TAYLOR, I.A. (1985). Application of DNA flow cytometry to paraffin-embedded archival material for the study of aneuploidy and its clinical significance. *Cytometry*, **6**, 327.

HIDDEMANN, W., SCHUMANN, J., ANDREEF, M. & 6 others. (1984). Convention on nomenclature for DNA cytometry. *Cancer Genet. Cytogenet.*, **13**, 181.

JOHANNESSEN, J.V., SOBRINHO-SIMOES, M., TANGEN, K.O. & LINDMO, T. (1981). A flow cytometric deoxyribonucleic acid analysis of papillary thyroid carcinoma. *Lab. Invest.*, **45**, 336.

KLEIN, M.J. & VALENSI, Q.J. (1976). Proximal tubular adenomas of the kidney with so-called oncocytic features: A clinicopathologic study of 13 cases of a rarely reported neoplasm. *Cancer*, **38**, 906.

KRAEMER, B.B., SRIGLEY, J.R., BATSAKIS, J.G., SILVO, E.G. & GOEPFEIT, H. (1985). DNA flow cytometry of thyroid neoplasms. *Arch. Otolaryngol.*, **111**, 34.

LIEBER, M.M., TOMERA, K.M. & FARROW, G.M. (1981). Renal oncocytoma. *J. Urol.*, **125**, 481.

VINDELØV, L.L., CHRISTENSEN, I.J. & NISSEN, N.I. (1983). A detergent-trypsin method for the preparation of nuclei for flow cytometric DNA analysis. *Cytometry*, **3**, 323.

WATSON, R.G., BRENNAN, M.D., GOELLNER, J.R., VAN HEERDEN, J.A., McCONAHEY, W.M. & TAYLOR, W.F. (1984). Invasive Hurthle cell carcinoma of the thyroid. *Mayo Clin. Proc.*, **59**, 851.